# Supplementary material for: In Vitro and In Silico Studies for the Identification of Potent Metabolites of Some High-Altitude Medicinal Plants from Nepal Inhibiting SARS-CoV-2 Spike Protein
Source: Molecules. 2022 Dec 15;27(24):8957. doi: 10.3390/molecules27248957 (PMC9786757; doi:10.3390/molecules27248957)
Supplement: Supplementary file 1 [file molecules-27-08957-s001.zip › molecules-1943010-supplementary.pdf]

Supplementary Materials for

In Vitro and In Silico Studies for the Identification of Potent Metabolites of Some High-Altitude Medicinal Plants from Nepal Inhibiting SARS-CoV-2 Spike Protein

Saroj Basnet [1,†], Rishab Marahatha [2,3,†], Asmita Shrestha [2,†], Salyan Bhattarai [4], Saurav Katuwal [2], Khaga Raj Sharma [2], Bishnu P. Marasini [5], Salik Ram Dahal [3,6], Ram Chandra Basnyat [2], Simon G. Patching [7,*] and Niranjan Parajuli [2,*]

[1] Center for Drug Design and Molecular Simulation Division, Kathmandu 44600,
[2] Central Department of Chemistry, Tribhuvan University, Kathmandu 44618, Nepal
[3] Department of Chemistry, Oklahoma State University, Still Water, OK 74078, USA
[4] Paraza Pharma, Inc., 2525 Avenue Marie-Curie, Montreal, QC H4S 2E1, Canada
[5] Nepal Health Research Council, Ramshah Path, Kathmandu 44600, Nepal
[6] Oakridge National Laboratory, Bethel Valley Rd, Oak Ridge, TN 37830, USA
[7] Independent Researcher, Leeds LS2 9JT, UK
* Correspondence: sgp_uk2000@yahoo.co.uk (S.G.P.); niranjan.parajuli@cdc.tu.edu.np (N.P.)
† These authors contributed equally to this work.

Table S1. Measurements of absorbance at 450 nm and calculation of % of hACE2 bound to the S1-RBD, detected by an anti-Human HRP antibody and TMB. (n=3).

S.N	Concentration (mg/mL)	Absorbance (mean)	% of hACE2 bound to the S1-RBD (mean)
**1.**	0.0000000	0.643	100.00000
**2.**	0.0390625	0.539	83.82582
**3.**	0.0781250	0.519	80.71540
**4.**	0.1562500	0.489	76.04977
**5.**	0.3125000	0.437	67.96267
**6.**	0.6250000	0.376	58.47589
**7.**	1.2500000	0.323	50.23328
**8.**	2.5000000	0.275	42.76827
**9.**	5.0000000	0.212	32.97045

Table S2. GOLD fitness score, binding free energy, and protein-ligand interactions of natural compounds with the S1-RBD region (6M0J).

Compound	GOLD Fitness Score	Binding free energy (ΔG_{bind}) (Kcal/mol)	Interacting residues	Bond Length (Å)
Cordifolioside-B (2)	43.06	−9.3	Thr430	2.8
			Phe515	2.2
Cordioside (3)	40.64	−7.7	Thr430	2.6/3.7
			Glu516	2.3
			Leu517	2.0/2.9
Cordifolide-A (4)	47.85	−8.9	Asp428	2.2
			Thr430	2.9
Cordifolide-B (5)	44.45	−9.7	Asp428	2.0
			Thr430	3.0
Cordifolide-C (6)	42.63	−9.8	Arg355	3.1
			Asp428	2.1

Tinosporaside (7)	39.92	−9.3	Thr430<br>Asp428	3.5<br>2.2
Tinosporiside (8)	38.38	−8.4	Arg355	2.9
Amritoside-A (9)	41.26	−6.8	Thr430<br>Glu516	2.9<br>2.2
Amritoside-B (10)	50.08	−6.9	Asp428<br>Leu517<br>Glu516	2.4<br>3.5<br>2.2
Amritoside-C (11)	41.70	−7.2	Glu516	2.2
Amritoside-D (12)	40.62	−7.3	Glu516	1.9
Palmitoside-F (13)	46.82	−8.8	Arg355	3.2
Palmitoside-G (14)	50.80	−21.23	Asp428	1.9/2.1

Table S3. Residues present in the active site and their RMSF values (Å) in the S1-RBD region.

Residue	Cordifolioside-A
333	0.3766
334	0.1627
335	0.1254
336	0.0966
337	0.0938
338	0.0869
339	0.0981
340	0.0921
341	0.073
342	0.0754
343	0.0856
344	0.0959
345	0.137
346	0.1225
347	0.081
348	0.0636
349	0.0604
350	0.0588
351	0.0625
352	0.0653
353	0.0598
354	0.0615
355	0.0624
356	0.0609
357	0.0686
358	0.0675
359	0.082
360	0.0936
361	0.0906
362	0.1027
363	0.1138
364	0.1219
365	0.1296
366	0.1833
367	0.1865
368	0.147

369	0.1846
370	0.2354
371	0.215
372	0.1858
373	0.1905
374	0.1277
375	0.1226
376	0.1021
377	0.0898
378	0.0765
379	0.0723
380	0.0784
381	0.0914
382	0.0902
383	0.1042
384	0.1351
385	0.1563
386	0.1378
387	0.1221
388	0.1317
389	0.1269
390	0.1043
391	0.0815
392	0.0655
393	0.0768
394	0.0723
395	0.0556
396	0.054
397	0.0497
398	0.0489
399	0.0494
400	0.0462
401	0.0487
402	0.0524
403	0.0672
404	0.0796
405	0.0867
406	0.0706
407	0.0694
408	0.0847
409	0.0793
410	0.0656
411	0.069
412	0.0787
413	0.112
414	0.0899
415	0.0895
416	0.0893
417	0.0868
418	0.0717
419	0.0697

420	0.0745
421	0.0719
422	0.0644
423	0.0592
424	0.0656
425	0.0712
426	0.0812
427	0.0919
428	0.0878
429	0.0745
430	0.0742
431	0.063
432	0.0575
433	0.0582
434	0.0579
435	0.0617
436	0.0624
437	0.0643
438	0.0631
439	0.0752
440	0.0929
441	0.0843
442	0.0681
443	0.0773
444	0.1006
445	0.1181
446	0.1258
447	0.1056
448	0.0808
449	0.0882
450	0.0877
451	0.07
452	0.0693
453	0.0675
454	0.0686
455	0.0821
456	0.0983
457	0.099
458	0.108
459	0.0972
460	0.0872
461	0.082
462	0.0841
463	0.0789
464	0.0778
465	0.0799
466	0.0775
467	0.0749
468	0.0781
469	0.0895
470	0.107

471	0.1236
472	0.1143
473	0.2459
474	0.3285
475	0.5356
476	0.5795
477	0.5066
478	0.4137
479	0.3224
480	0.2261
481	0.2885
482	0.3086
483	0.3114
484	0.3539
485	0.3893
486	0.3112
487	0.2814
488	0.179
489	0.1208
490	0.1065
491	0.0825
492	0.0845
493	0.0835
494	0.0797
495	0.0706
496	0.0856
497	0.0749
498	0.0854
499	0.1089
500	0.1363
501	0.1318
502	0.1317
503	0.1282
504	0.1094
505	0.0873
506	0.0643
507	0.0535
508	0.0503
509	0.0483
510	0.0471
511	0.0472
512	0.0471
513	0.0517
514	0.0586
515	0.0665
516	0.0756
517	0.098
518	0.1214
519	0.1545
520	0.1523
521	0.1352

522	0.1042
523	0.1003
524	0.076
525	0.1044
526	0.1763

Table S4. ADMET properties of the major compounds of *T. cordifolia* (1-14) by pkCSM server.

	Parameter	1	2	3	4	5	6	7	8	9	10	11	12	13	14
**Absorption**	Water solubility (log mol/L)	−1.965	−2.112	−2.88	−3.504	−3.393	−3.393	−3.079	−3.073	−4.633	−2.704	−3.823	−3.757	−4.819	−2.936
	Caco2 permeability (log Papp 10-6 cm/s)	−0.368	−0.613	−0.408	−0.307	0.591	0.591	−0.348	−0.386	1.077	−0.237	1.178	1.267	0.485	−0.404
	Intestinal absorption (% absorbed)	31.087	46.863	52.66	49.384	69.799	69.799	59.506	54.034	94.917	43.031	92.024	95.383	100	57.067
	Skin permeability (log Kp)	−2.735	−2.737	−2.743	−2.737	−2.735	−2.735	−2.919	−2.784	−3.485	−2.735	−3.38	−3.538	−3.203	−2.784
**Distribution**	VDss (Human, log L/Kg)	0.009	−0.293	−0.515	0.02	−0.003	−0.003	−1.01	−0.673	0.114	−0.73	0.008	0.151	−0.079	−0.611
	BBB Permeability (logBB)	−1.636	−1.884	−1.682	−1.836	−1.435	−1.435	−1.553	−1.348	−0.364	−1.467	−0.653	−0.233	−0.922	−1.384
	CNS Permeability (log PS)	−5.353	−4.946	−4.934	−4.925	−4.591	−4.591	−5.079	−4.194	−2.906	−3.564	−3.017	−2.966	−3.05	−4.273
**Metabolism**	CYP1A2	NO	NO	NO	NO	NO	NO	NO	NO	NO	NO	NO	NO	NO	NO
	CYP2C19	NO	NO	NO	NO	NO	NO	NO	NO	NO	NO	NO	NO	NO	NO
	CYP2C9	NO	NO	NO	NO	NO	NO	NO	NO	NO	NO	NO	NO	NO	NO
	CYP2D6	NO	NO	NO	NO	NO	NO	NO	NO	NO	NO	NO	NO	NO	NO
	CYP3A4	NO	NO	NO	NO	NO	NO	NO	NO	NO	NO	YES	NO	YES	NO
**Excretion**	Renal OCT2 substrate clearance	NO	NO	NO	NO	NO	NO	NO	NO	NO	NO	NO	NO	NO	NO
	Total Clearance (logml/min/kg)	0.846	0.768	0.857	0.541	0.574	0.574	0.637	0.605	1.247	0.978	1.058	1.021	0.878	0.755
**Toxicity**	Ames Toxicity	NO	NO	NO	NO	NO	NO	NO	NO	NO	NO	NO	NO	NO	NO
	Hepatotoxicity	NO	NO	NO	NO	NO	NO	NO	NO	NO	YES	YES	NO	NO	NO
	LD_{50}	2.778	2.949	3.78	3.471	3.003	3.354	3.521	3.113	2.988	2.77	3.718	3.57	3.844	3.354

Value Range : LogS (Solubility): Optimal (higher than -4log mol/L) , **Papp (Caco-2 Permeability):** Optimal (higher than -5.15 Log unit or -4.70 or -4.80) , **HIA (Human Intestinal Absorption)**: >30% Perfectly absorbed , **VD (Volume Distribution)**: Optimal (0.04-20 L/Kg) , **BBB (Blood Brain Carrier):** (BB ratio >=0.1: BBB+ ; BB ratio <0.1: BBB-) , **Total Clearance:** >15 ml/min/kg: High; 5 ml/min/kg< CL< 15ml/min/kg: Moderate; <5 ml/min/kg: Low, **LD_{50}(LD_{50} of acute toxicity):** High-toxicity: (1-50 mg/kg); Modarate-toxicity: (51-500 mg/kg); Low-Toxicity: (501-5000 mg/kg).

Table S5. Prediction of toxicity of secondary metabolites inhibiting metabolic enzymes using ProTox-II.

Compounds	LD_{50} mg/Kg	Toxicity class	Active target	Probability
Cordifolioside-A (1)	4000	5	Immunotoxicity	0.99
Cordifolioside-B (2)	4000	5	Immunotoxicity	0.99
Cordioside (3)	274	3	Immunotoxicity	0.96
			Mitochondrial Membrane Potential (MMP)	0.76
Cordifolide-A (4)	244	4	Immunotoxicity	0.99
			Mitochondrial Membrane Potential (MMP)	0.71
			P53	0.74
Cordifolide-B (5)	500	4	Immunotoxicity	0.99
			Cytotoxicity	0.79
			Alpha Estrogen receptor ligand- binding domain	0.76
			Mitochondrial Membrane Potential (MMP)	0.81
Cordifolide-C (6)	500	4	Immunotoxicity	0.99
			Cytotoxicity	0.79
			Alpha Estrogen receptor ligand- binding domain	0.76
			Mitochondrial Membrane Potential (MMP)	0.81
Tinosporaside (7)	274	3	Immunotoxicity	0.99
Tinosporiside (8)	280	3	Immunotoxicity	0.96
Amritoside-A (9)	274	3	Immunotoxicity	0.97
Amritoside-B (10)	244	3	-	-
Amritoside-C (11)	555	4	Immunotoxicity	0.96
			Aromatase	0.79
			Mitochondrial Membrane Potential (MMP)	0.83
			P53	0.72
Amritoside-D (12)	274	3	Immunotoxicity	0.96
			Estrogen Receptor Alpha (ER)	0.75
			PPAR-gamma	0.73
			Mitochondrial Membrane Potential (MMP)	0.77
Palmitoside-F (13)	310	3	Immunotoxicity	0.99
Palmitoside-G (14)	274	3	Immunotoxicity	0.98

Table S6. List of major natural compounds of *T. cordifolia*.

Pharmacological properties	Major natural compounds selected for the study
	Cordifolioside A(1)
	Cordifolioside-B(2)
	Cordioside(3)
1. Anti-SARS (2)	Cordifolide-A(4)
2. Anti-Influenza (3)	Cordifolide-B(5)
3. Anti-Herpes (4)	Cordifolide-C(6)
4. Anti-HIV (5)	Tinosporaside(7)
5. Anti-bacterial (6)	Tinosporiside(8)
6. Anti-fungal (7)	Amritoside-A(9)
7. Anti-malarial (8)	Amritoside-B(10)
8. Anti-inflammatory (9)	Amritoside-C(11)
	Amritoside-D(12)
	Palmitoside-F(13)
	Palmitoside-G(14)

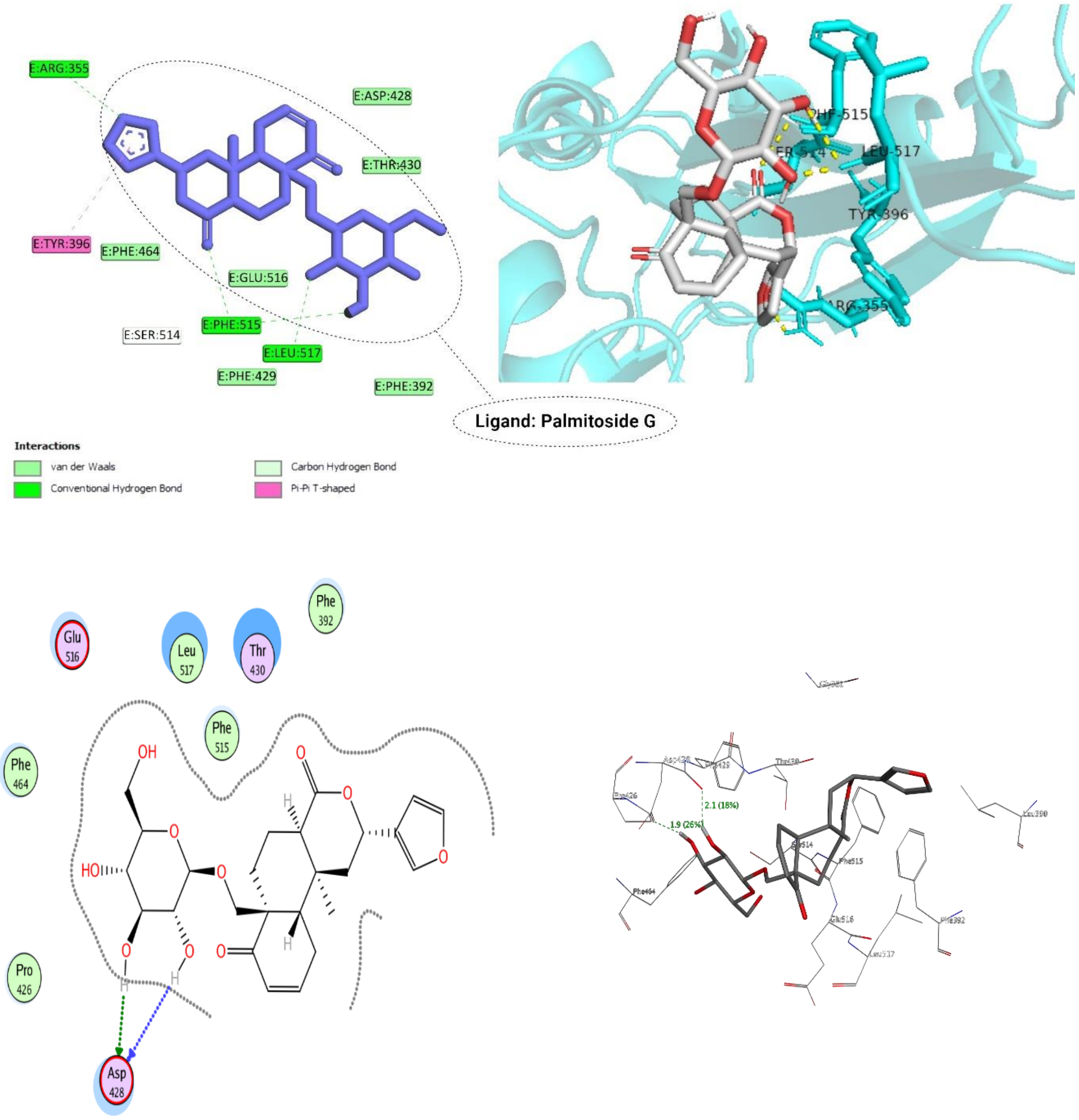

Figure S1. Binding interactions and 2D (left) and 3D (right) interaction of palmitoside-G (14) with S1-RBD.
